# The relevance of nanoscale biological fragments for ice nucleation in clouds

**DOI:** 10.1038/srep08082

**Published:** 2015-01-28

**Authors:** D. O′Sullivan, B. J. Murray, J. F. Ross, T. F. Whale, H. C. Price, J. D. Atkinson, N. S. Umo, M. E. Webb

**Affiliations:** 1 Institute for Climate and Atmospheric Science, School of Earth & Environment, University of Leeds, UK; 2 School of Chemistry and Astbury Centre for Structural Molecular Biology, University of Leeds, UK; 3 Now at Institute for Atmospheric and Climate Science, Universitaetstr. 16, ETH Zurich, Switzerland

## Abstract

Most studies of the role of biological entities as atmospheric ice-nucleating particles have focused on relatively rare supermicron particles such as bacterial cells, fungal spores and pollen grains. However, it is not clear that there are sufficient numbers of these particles in the atmosphere to strongly influence clouds. Here we show that the ice-nucleating activity of a fungus from the ubiquitous genus *Fusarium* is related to the presence of nanometre-scale particles which are far more numerous, and therefore potentially far more important for cloud glaciation than whole intact spores or hyphae. In addition, we quantify the ice-nucleating activity of nano-ice nucleating particles (nano-INPs) washed off pollen and also show that nano-INPs are present in a soil sample. Based on these results, we suggest that there is a reservoir of biological nano-INPs present in the environment which may, for example, become aerosolised in association with fertile soil dust particles.

The formation of ice in mixed-phase clouds, where both supercooled water droplets and ice coexist, impacts upon precipitation, cloud lifetimes and radiative properties^1^. Ice formation is often catalysed by the presence of ice-nucleating particles (INPs) at temperatures much warmer than required for homogeneous nucleation in pure water droplets. Due to their impact on cloud properties, determination of the number and identity of particles which can act as INPs in the atmosphere has seen a resurgence in interest from researchers attempting to understand the mechanisms by which supercooled water droplets are transformed into ice^2^.

While inorganic INPs are thought to be of critical importance in the initiation of ice at temperatures below approximately −20°C, these species (most notably mineral dusts) are unlikely to be sufficiently active above about −15°C to influence clouds^3^,^4^. At warmer temperatures, where secondary ice production mechanisms such as the Hallet-Mossop process are active, the only classes of aerosol particles known to exhibit a substantial ability to nucleate primary ice are biogenic in origin^5^. However, the atmospheric concentration of whole and intact airborne biogenic particles (e.g. bacteria cells, pollen grains and fungal spores) tends to be too small to significantly influence cloud glaciation on a global scale, although their relevance may increase close to emission sources or in regions without other INP sources^6^,^7^. Consequently, observations of glaciation in clouds with cloud top temperatures above −15°C (see for example Kanitz et al.^8^) have remained difficult to explain based on both the relatively low activity of inorganic INPs and the relatively few intact biological entities capable of nucleating ice^3^.

It is known that the ice-nucleating sites on intact biogenic INPs are far smaller than the intact cells, grains or spores themselves, and in some cases can be entirely harboured on separable fragments^9^,^10^,^11^,^12^. For example, Augustin *et al.* have estimated that for birch pollen, a single grain can possess tens of thousands of ice-nucleating nano-scale particles, each of which can be readily extracted from the parent grain by contact with water^13^. It has also been shown that INPs in precipitation and leaf litter can be nanometre in scale^14^,^15^, which is consistent with the recent finding that INPs from the soil fungus *Mortierella alpina* are also nanometer in scale^16^. However, the current body of literature on biogenic INPs mainly focuses on whole, intact biological entities such as bacteria, fungal spores or pollen grains, and questions on whether smaller biogenic fragments can participate in atmospheric ice nucleation remain open.

In this paper we hypothesise that biological, nanometer scaled INPs which are bound to other atmospheric particulates are important for cloud glaciation; this hypothesis is illustrated in Figure 1. As noted above, the concentration of intact bacteria, fungal spores and pollen are too low in many locations to impact cloud glaciation, but it is possible that the concentration of biological nano-INPs is much greater. Nano-scale particulates are unlikely to be emitted to the atmosphere directly, but instead can be associated with larger particles such as soil dust particles. Biological species residing in, or deposited to soil, such as fungi, bacteria and pollen, may produce nano-INPs which could attach to soil particles and subsequently become aerosolised. Indeed, recent studies examining the ice-nucleating abilities of agricultural soil dusts have shown that unidentified components of the soil organic matter can exhibit potent ice-nucleating activities^17^,^18^,^19^,^20^. Similarly, recent studies examining cloud ice crystal residues in mixed phase clouds impacted by dust aerosols transported 1000 s of miles have found that biogenic matter internally mixed with mineral dusts play an important role in ice formation within supercooled clouds^21^,^22^.

To explore this hypothesis, we examine the ice-nucleating activities of nano-particles associated with biological materials including a soil-borne plant pathogenic fungus from the widely distributed genus *Fusarium*, birch (*Betula pendula*) pollen and a soil sample. Through a combination of filtration and size exclusion chromatography we demonstrate that the entities responsible for nucleation in *Fusarium avenaceum* are nano-scale species which are readily extractable into water. In addition it is shown that individual pollen grains can harbour on the order of 10^4^ distinct ice-nucleating nano-particles capable of inducing nucleation at relatively warm temperatures (T > −18°C). We also demonstrate that nano-INPs can also be found in soils, and exhibit comparable thermal instabilities to known proteinaceous INPs, such as those we find in *Fusarium avenaceum*.

## Results

### The size of fungal ice nucleating particles

**Figure 2:**
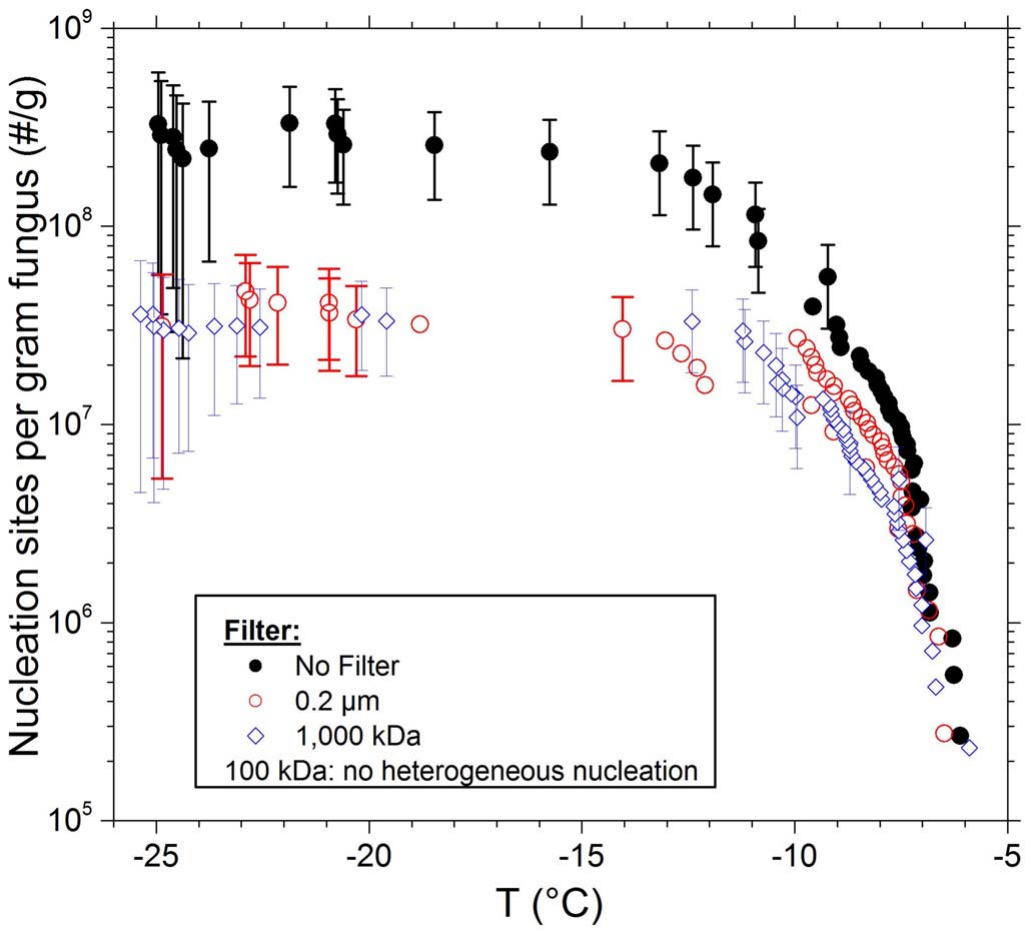
Ice nucleation activity of a sample of the fungus *Fusarium avenaceum*. The activity of the mycelium suspended in water is shown, along with the activities of the 0.2 μm, 1,000 kDa and 100 kDa filtrates. The number of active sites is normalized to the fresh mass of the whole fungus used to prepare the suspensions. Upon filtration through a 100 kDa filter the activity is found to drop below the baseline for the measurement, below which point nucleation events cannot be distinguished from nucleation by impurities in the Milli-Q® purified water. For clarity, representative error bars are only shown for a selection of data points.

By suspending known quantities of fungal (*F. avenaceum*) mycelium in microlitre-sized droplets and cooling them while monitoring for freezing events, we determined the cumulative number of active nucleation sites as a function of temperature (referred to as a cumulative nucleus spectrum). The number of active sites is reported per unit mass of the mycelium suspended in the droplets (see methods). To facilitate observation of ice nucleation at colder temperatures, experiments were also performed on suspensions which were serially diluted by factors of 100 and the results are shown in Figure 2. Droplets containing unfiltered fungal mycelium started to freeze at around −6°C. This indicates that *F. avenaceum* contains highly active INPs, consistent with previous reports^10^,^23^. We also show the ice nucleation spectra for water droplets containing suspended mycelium which were subsequently passed through a sequence of filters. Heterogeneous ice nucleation at around −6°C continued to occur in the droplets following 0.2 μm filtration, consistent with Pouleur *et al*.^10^. Suspensions were then passed through a membrane filter with a molecular weight cutoff of 1000 kDa; this would correspond to a smooth spherical particle with a diameter (*d*_smooth sphere_) of 13 nm for the average density of a typical protein (we take a protein density of 1.37 g cm^−3^ from Erickson^24^ who discuss the relationships between different metrics of protein size and shape). Surprisingly, these samples contained the same concentration of INPs as the 0.2 μm filtrate. When the filtrate was passed through a filter with a nominal molecular weight cut-off of 100 kDa (*d*_smooth sphere_ ~6 nm), the ice-nucleating activity was dramatically reduced, falling below the limit of detection for the assay determined by background freezing of the Milli-Q® purified water due to impurities (see methods). These results show that the INPs produced by *F. avenaceum* can function independently of fungal cells, are easy to remove from the parent fungus and are nanometre in scale.

**Figure 3:**
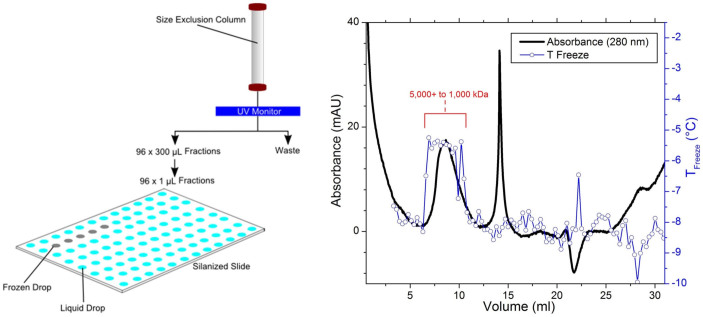
Size exclusion chromatography combined with an ice nucleation assay to study the size of INPs in the fungus *Fusarium avenaceum*. (Left) Experimental schematic of the fractionation procedure. Initially, 96 × 300 μL fractions are collected from the Superose 6 10/300 GL column. A droplet freezing experiment is then performed using a 1 μL droplet from each of the fractions. (Right) UV absorbance (black trace) and freezing temperature of eluted fractions (blue trace) after passing through a Superose 6 column. The appearance of elevated nucleation temperatures can be seen concurrent to elevated absorbance in the eluate at flow through volumes of 6–11 mls (~1000–5000+ kDa).

To further examine the size of the ice-nucleating particles generated by *F. avenaceum*, the 1000 kDa filtrate was separated into 96 size-resolved fractions using size-exclusion chromatography (Figure 3, left). This technique separates suspended particles on the basis of size, with smaller particles being eluted at later times (or larger elution volumes). When the ice nucleating activity of the fractions was examined, those with higher nucleation temperatures were found to correspond to fractions exhibiting a peak in the 280 nm UV absorbance (Figure 3, right), consistent with the INPs being proteinaceous. While all fractions were found to freeze at temperatures above −11°C, the highest freezing temperatures observed can be seen to appear concurrent with a fully resolved broad feature on the chromatogram at short retention times (eluate volume 6–11 mls). Of note, while a species of this size (~1000–5000+ kDa; *d*_smooth sphere_ = 13–23+ nm) is larger than the nominal molecular weight cut-off of the filter applied prior to size exclusion chromatography, this is consistent with a finite distribution of pore sizes as is found in ultrafiltration membranes^25^.

There are several similarities between the nano-INPs produced by *F. avenaceum* and those from the well-known ice-nucleating γ−proteobacteria, such as *Pseudomonas syringae*. For instance, the activities of both are comparable. Furthermore, both are thought to contain proteins in the ice-nucleating active sites and are sensitive to heat treatment and enzymatic digestion with proteases^10^,^23^,^26^,^27^. However, there are also several key differences between fungal and bacterial ice-nucleators. For instance, the ice nucleating activity in bacterial proteins is related to the presence of membrane lipids in cells or cell fragments^28^. The activity of fungal INPs is not dependent on membrane lipids^29^. Furthermore, fungal INPs are more stable to pH and heat treatment than bacterial INPs^10^. These intrinsic differences between fungal and bacterial ice-nucleators suggest that the two may have arisen independently of each other in nature^30^.

### Ice nucleating nano-particles from pollen

**Figure 4:**
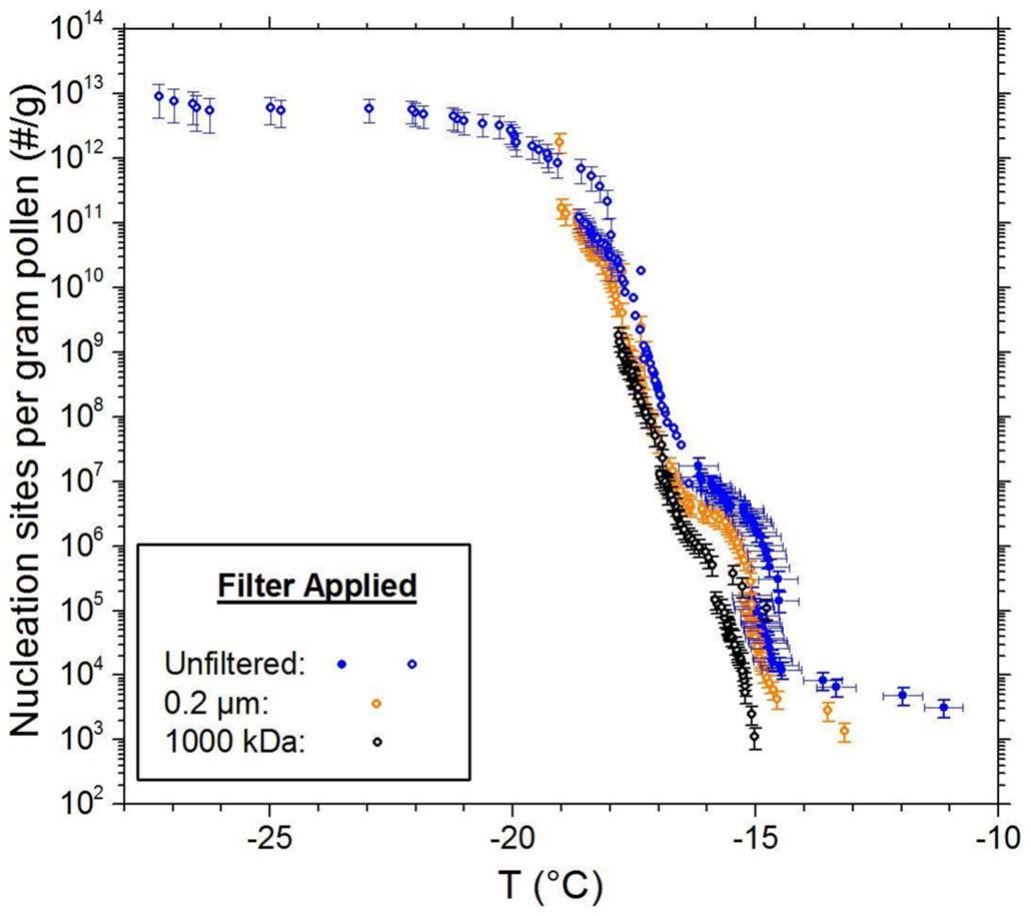
Cumulative nucleus spectra for pollen (*Betula pendula*) suspensions containing whole grains (blue symbols), 0.2 μm filtered suspensions (orange) and 1000 kDa filtered suspensions (black). The intial concentrations used to prepare the solutions was 2 wt. %. To examine nucleation at temperatures lower than achievable at this concentration, the suspensions were serially diluted. For the unfiltered data, data with a dilution factor of 10^4^ or greater are shown with open symbols to indicate where the expectation value for the number of grains per droplet drops below zero, and only species smaller than whole grains remain in suspension. Values are normalized to the original mass of pollen used. Most of the activity in the parent (unfiltered) suspension can be seen to remain after 0.2 μm filtration and ultrafiltration to 1000 kDa.

Another class of biogenic nano-INPs are derived from pollen^9^,^13^. In contrast to both bacterial and fungal ice nucleators, the ice-nucleating sites in pollens are not thought to contain proteins, but instead have been suggested to stem from carbohydrates^9^. Similar to what was observed for the *Fusarium* fungus studied here, contact with water is sufficient to separate a large proportion of the nano-INPs from the parent grains. Shown in Figure 4 are the cumulative nucleus spectra for suspensions initially containing birch pollen grains, along with data taken from the same solutions when passed through 0.2 μm and 1000 kDa filters. Starting with a concentration of 2 wt. %, the cumulative nucleus spectra were discerned by sequential 100-fold dilutions to enable observation of freezing events at increasingly lower temperatures. Only a minor loss of activity is observed on filtration to 0.2 μm and 1000 kDa, confirming that the INPs are nanometre in scale and easily separated from the pollen grains into water.

At temperatures below −18°C the characteristically sharp slope of the nucleation spectrum for pollen-derived INPs levels off (Figure 4). This behaviour is consistent with the loss of INPs owing to the high level of dilution (1/10^8^ of the original 2 wt. % concentration) used to assess nucleation in this regime. From this, the number of nano-INPs active at temperatures greater than −18°C (i.e. before they are removed by dilution) can be estimated as <10^12^ per gram from Figure 4. With ~1.2 × 10^8^ grains per gram for birch pollen^13^,^31^,^32^ this equates to 8 × 10^3^ INPs per grain, similar to the 2 × 10^4^ estimated by Augustin *et al.*^13^ for a birch pollen (*Betula pendula*) using a different approach.

### Nano-scaled ice nucleating particles in soils

**Figure 5:**
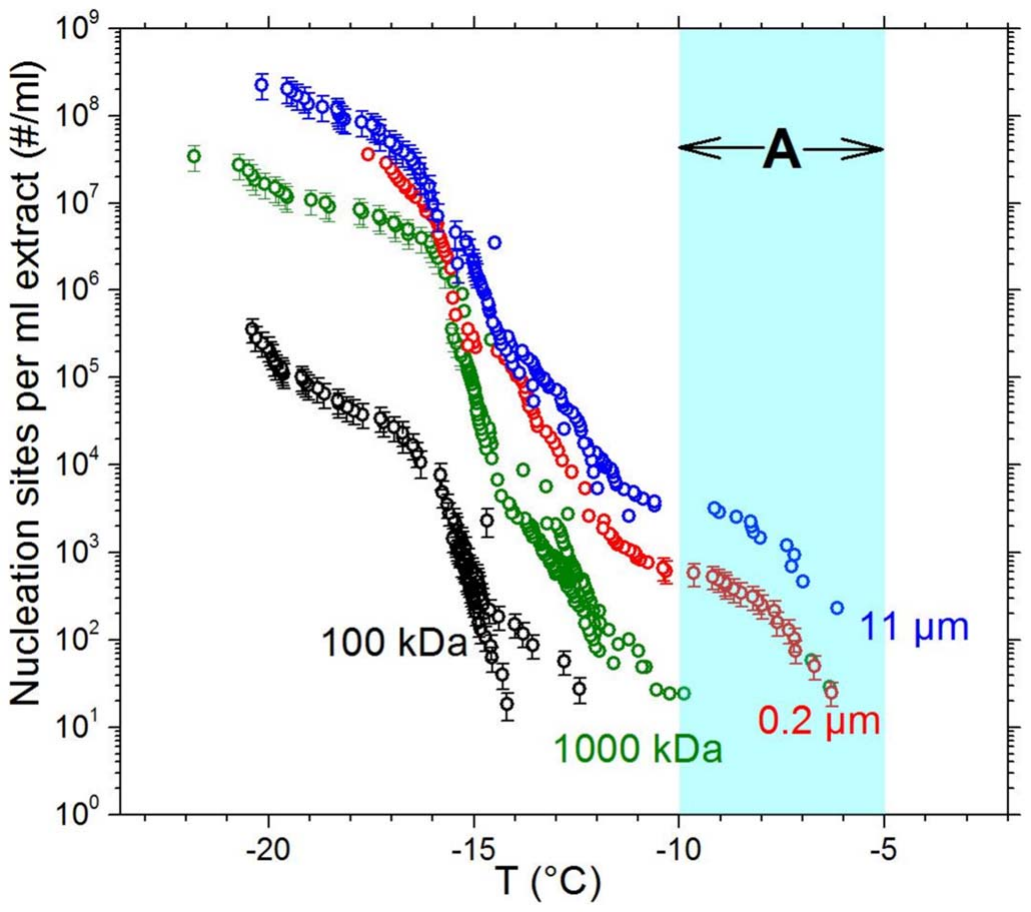
Cumulative nucleus spectra for multiple size fractions of soil particles dispersed in water. This plot shows the temperature dependence of the number of nucleation sites per millilitre of a 1:10 (mass soil:volume water) suspension of the soil which pass through each filter. The shaded region, labelled A, indicates the population of INPs which are active at temperatures above −10°C. For clarity, error bars are only shown for a selection of data points.

To examine whether biogenic nano-INPs can be found in natural samples containing decomposing organic matter, filter fractionation tests were also applied to a sample of soil collected in the United Kingdom. Similar to the samples of *F. avenaceum* and for birch pollen, nano-INPs were also observed to stimulate ice nucleation in aqueous soil suspensions (Figure 5). On successive filtration through 11 μm, 0.2 μm, 1000 kDa and 100 kDa filters, active INPs remained in the droplets. The profiles of each spectrum differ between the fractions, suggesting that there are multiple distinct populations of INPs present. For instance, in both the 11 μm and 0.2 μm fractions, particles capable of nucleating ice at relatively small supercoolings (above −10°C, labelled regime A in Figure 5) are present. On further filtration to 1000 kDa, this component of the cumulative nucleus spectrum is completely lost, suggesting that these ice nucleating sites, which are active at low supercoolings, are more frequently associated with particles larger than 0.2 μm. In addition, there is clearly a significant population of nano-INPs which pass through the 1000 kDa filter. The spectrum for the 1000 kDa fraction approaches those of the 0.2 and 11 μm fractions at about −16°C, suggesting that a substantial proportion of the activity in these larger fractions may be attributable to the presence of nano-INPs. Furthermore, there are also INPs which pass through a 100 kDa filter and are active below about −12°C (a 100 kDa smooth spherical protein would have a diameter of about 6 nm). This soil sample clearly contains a range of INP types with sizes ranging from microns to nanometres in scale.

**Figure 6:**
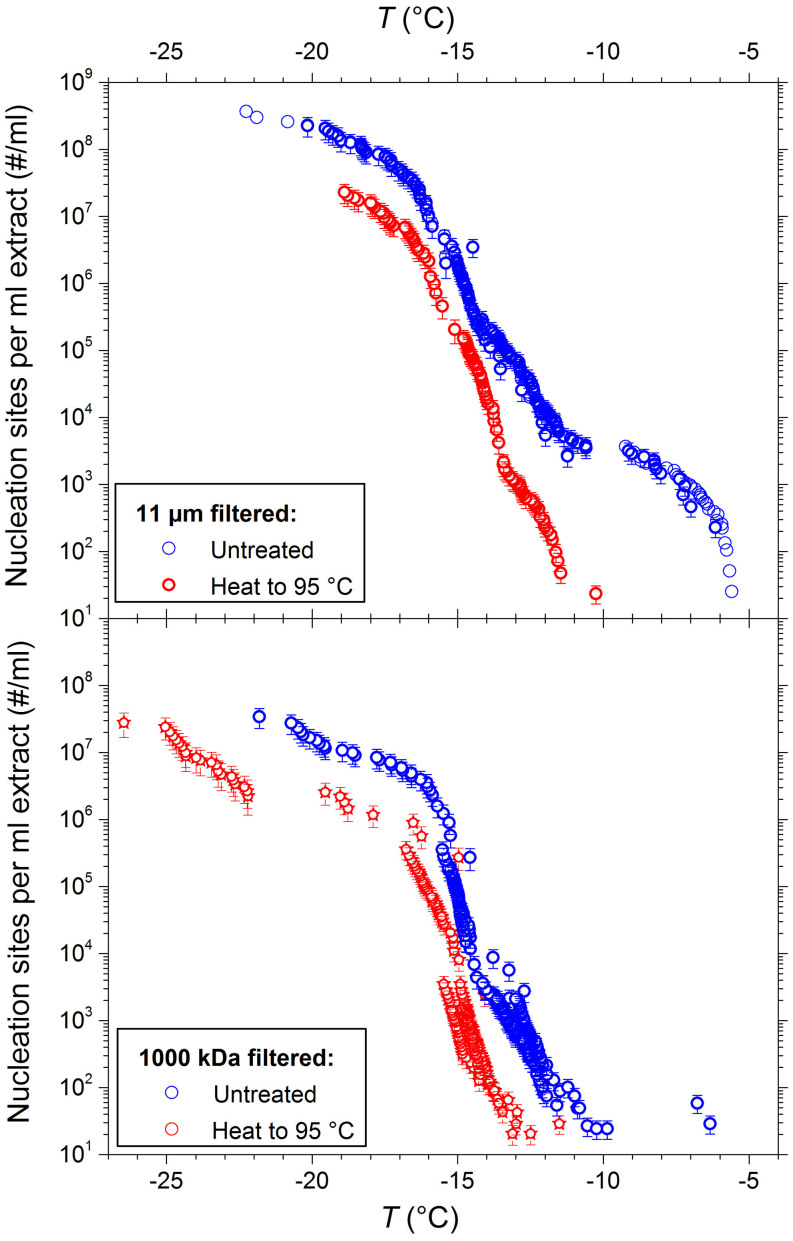
Cumulative nucleus spectra for a soil suspension diluted 1:10 which passes through an 11 μm and 1000 kDa filter. The number of nucleation sites present before (blue) and after (red circles) heating the suspension to 95°C for 45 minutes are shown. Sensitivity to heat treatment can be seen in both size fractions.

In order to examine whether the INPs in soils may be biogenic in origin, the 11 μm and 1000 kDa soil suspension filtrates were heat treated (Figure 6). Loss of activity upon heating to temperatures of 90°C or higher is a common qualitative test for the presence of proteinaceous INPs, such as are produced by *P. syringae* and *Fusarium* fungi^10^,^33^,^34^,^35^,^36^. Conversely, the carbohydrate based INPs associated with pollen^9^ or inorganic components of soil dust which exhibit ice-nucleating activities, such as microcline^37^, are unaffected by such treatment (see Supplementary Fig. S1 for the effects of heat treatment on microcline). Upon heat treatment, the population of INPs available to nucleate above −10°C in the 11 and 0.2 μm fractions are almost completely lost (the former illustrated in Figure 6, see Supplementary Fig. S3 for the analogous chart for the 0.2 μm fraction). This, combined with the fact that these efficient INPs do not pass through 1000 kDa filters is consistent with these nuclei originating from proteins associated with larger particles, such as mineral dust or subcellular fragments of ice nucleating bacteria (which can also exhibit ice nucleation activity^38^).

While the most efficient heat sensitive INPs are removed by filtration to 1000 kDa, other less efficient INPs are not removed yet are also sensitive to heat treatment (Figure 6), suggesting the presence of nano-scaled, likely proteinaceous, INPs. Additionally, the thermal stabilities of INPs active at each temperature was found to vary across the size fractions examined. For instance, while at −13°C heat treatment of the 1000 kDa fraction is found to lead to an approximately 50-fold reduction in the ice nucleating activity, at −15°C only an approximately 4-fold reduction was observed. The above findings highlight the complexity of ice nucleators in naturally occurring samples, where multiple discrete populations of INPs can coexist, each with active sites of different efficiencies situated on particles of vastly different sizes.

## Discussion

The characterisation of particles responsible for ice nucleation in the atmosphere is of central importance to understanding glaciation in mixed phase clouds. Determining the species responsible for ice nucleation in complex mixtures such as soil dusts requires characterising the ice-nucleating abilities of trace components. Here, we have shown that nano-scaled proteinaceous residues from the soil-borne fungus *F. avenaceum* can exhibit exceptional ice-nucleating abilities, and have quantified the ice nucleating activities of suspendable nano-INPs from a birch pollen. These nano-INPs are amongst both the most active and the smallest INPs which have been examined to date. The discovery that some INPs produced by fungi are of nanometre scale is very exciting because it raises the possibility that there could be a significant amount of this material in the atmosphere, most likely associated with other aerosol particles, which has not been accounted for in models. Furthermore, we show that nano-INPs are also present in a natural soil sample containing organic matter in various stages of decomposition. These findings help to substantiate the hypothesis that biogenic nano-INPs can play important roles in determining the ice-nucleating activity of environmental samples, particularly at temperatures above −20°C.

Past research examining the links between the exceptional ice-nucleating abilities of biogenic aerosol particles and cloud glaciation have focused on the impacts of intact bioaerosol types (e.g. certain bacteria, fungal spores and pollens)^3^,^4^,^6^,^39^. However, the observation that readily-extractable, nanometre-scale fragments of biogenic ice nucleators are efficient INPs suggests that these species may impact upon cloud glaciation via mechanisms which have yet to be explored in detail. For instance, aerosolized dusts are often contaminated with organic matter^21^,^22^,^40^, which may contain biogenic nano-INPs that can radically alter the ice-nucleating behaviour of the dusts. Given the relatively low ice-nucleating ability of organic-free mineral dusts at temperatures above −15°C^37^, organic residues which become mobilized together with soil dusts may play an integral role in cloud glaciation.

The results presented here suggest that in order to increase the accuracy of our current model simulations of atmospheric ice nucleation processes, biogenic species other than supermicron-sized particles should be considered. However, deficiencies currently exist in the basic knowledge required to accurately assess the impacts of biological nano-INPs on cloud ice formation. These deficiencies include our knowledge of the source strengths and global distribution of nano-INPs. There are also unresolved questions on the stability of biogenic nano-INPs towards chemical and physical processing in soils and when suspended in the atmosphere.

While producing inventories of the species responsible for ice-nucleating activity in complex mixtures such as soil and constraining the impacts of such particles on atmospheric ice nucleation processes is likely to be a complex undertaking, there are several factors which suggest that such a task is achievable. Relatively simple tests such as filtration, heat treatment and enzymatic digestion can be used for the rapid screening of biogenic nano-INPs in natural aerosol samples. In such a manner, datasets of the abundance of biogenic ice nucleators can be established, and their regional importance quantified. Links between the net amount of biogenic nano-INPs and governing variables (e.g. climate type, land use etc.) can then be examined, allowing for the global evaluation of biogenically derived nano-INPs.

## Methods

### Sample Preparation

Freeze-dried cultures of *F. avenaceum* were obtained from the Commonwealth Agricultural Bureaux International (CABI). The *F. avenaceum* were plated on potato dextrose sucrose agar and incubated for 28 days in the dark at 20°C. The colonies produced were ca. 4 cm in diameter, and varied in colour from white to pink. 1 mg of the mycelium was then subsequently scraped off the agar and resuspended in 10 ml of Milli-Q® grade water. No other steps to induce cell lysis were taken. The suspension was then filtered first through a 0.2 μm cellulose acetate filter (Sartorius Stedim®, Minisart 16534), followed by a polyethersulfone centrifuge filter (molecular weight cutoff = 1000 kDa, Sartorius, Vivaspin 6 VS0661) and regenerated cellulose filter (molecular weight cutoff = 100 kDa, Millipore, Amicon Ultra, UFC910008). The 1000 kDa filtrate was further fractionated by size exclusion chromatography using a Superose 6 10/300 GL column capable of separating proteins from 5,000–5,000,000 kDa in size. The fractionation was conducted in pure water (not in buffer) to allow for ice nucleation measurements. Protein elution was monitored via UV absorbance of the eluate at 280 nm.

The pollen used in this study was collected from wild Silver birch (*Betula pendula*) trees growing in South Moravia, Czech Republic, and was supplied by Pharmallerga®. The batch (BETP.1310) was certified to contain <0.2% spores, <0.1% other foreign pollen and the pollen was not defatted. To extract the nano-INPs, 1 g of the dried pollen was suspended in 50 ml water (2 wt. %), shaken and allowed to settle overnight in a refrigerator. After 12 hours, the pollen solution was shaken again, and then filtered, first through a 11 μm Nylon net filter (Millipore) and then through a 0.2 μm cellulose acetate filter. To obtain the 1000 kDa ultrafiltrate, Sartorius Vivaspin Ultrafilter devices as described above were employed.

A woodland soil sample was collected from the top 8 cm at a site in Cumbria in North West England (coordinates: 54.445976, −2.959535). 10 g of the soil (<2 mm) was suspended in 100 ml of water, and sonicated for 3 minutes to disperse the sample. A 45 ml aliquot of the sample was then poured into a polypropylene centrifuge tube, and left to settle for 60 s to allow larger particles to settle out. The supernatant was withdrawn from the top 4 cm of the vial for filtration. For the 11 μm filtration, the sample was vacuum filtered through a hydrophilic nylon net filter (Millipore, NY1004700). Filtration to 0.2 μm was conducted using a cellulose acetate filter. Ultrafiltration to yield 1000 kDa and 100 kDa fractions was conducted using Millipore Amicon Ultra and Sartorius Vivaspin centrifuge filters as outlined above. Heat treatment of the soil samples to test for thermally-labile INPs was conducted by heating a sealed Eppendorf tube containing 1 ml of the suspension to 95°C for 45 minutes. This test, which is commonly used as a qualitative assay for the presence of proteinaceous INPs^18^,^33^,^34^,^41^, was also performed on samples of the ice-nucleating mineral dusts potassium feldspar polymorph microline and NX illite. No significant differences were found in the ice-nucleating activities of these minerals after the same heat treatment was applied (Supplementary Fig. S1).

### Drop freezing experiments

The microlitre drop-freezing experiments have been described in detail previously^18^,^37^,^42^, and so are only briefly discussed here. For the experiments examining microlitre-sized droplets, a Stirling-engine-chilled aluminium stage (Grant-Asymptote, EF600) was used to chill the underside of a siliconised glass slide (Hampton Scientific, HR3-231) which supported the droplets, which were 1 μl in volume. The headspace above the droplets was encapsulated by a Perspex shield and gently purged using 200 cm^3^ min^−1^ of nitrogen gas to prevent unwanted condensation. The cooling rate employed for all experiments was 1 K min^−1^. Freezing events were monitored using a digital camera and later the video was analysed frame by frame to determine the onset of nucleation. The temperature error associated with the thermocouple was estimated as ±0.4 K. All filters employed were tested for the presence of heterogeneous INPs which could interfere with the measurements. In each case, the freezing profile indicated that droplet nucleation took place at temperatures lower than was found for Milli-Q® purified water (Supplementary Fig. S2), the freezing of which is influenced by nucleation due to the supporting substrate or impurity particles. To characterise the activities of the INPs in suspension, the undiluted suspensions as described above were initially analysed, followed by further experiments with serial dilutions in steps of factors of 10 or 100 fold dilution until the activities could not be differentiated from that of the Milli-Q® purified water. The maximum dilutions for *F. avenaceum*, *Betula pendula* pollen and the woodland soil extract examined were 1/100, 1/10^8^ and 1/10^4^, respectively.

### Data analysis

Using a time-independent (singular) analysis of the fraction of droplets frozen at a particular temperature^3^,^43^, *F(T)*, the cumulative number of nucleation sites active at a given temperature per unit volume of water, *K*(*T*), can be determined by:
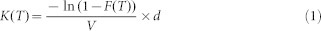
where *V* is the droplet volume in cm^3^. The factor *d* is the dilution of the droplets relative to the initial undiluted suspension. Accordingly, by diluting the suspension, the ice-nucleating activity can be probed at lower temperatures than is observable in droplets of the initial concentration. For a known amount of nucleant per unit volume, the cumulative nucleation spectrum can be expressed relative to the mass of material suspended in the droplet via:
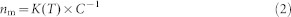
where *C* is the concentration of the parent particles in g/cm^3^. Uncertainties in the calculation of the number of nucleation sites are calculated by propagating those stemming from the concentration and droplet volume together with uncertainties which arise due to background freezing of the Milli-Q® water. In all experiments on Milli-Q® water where no nuclei were added, freezing occurred at temperatures higher than expected due to homogeneous nucleation^44^. The number of heterogeneous nucleation sites stemming from contaminants in the Milli-Q® purified water was determined from a compilation of 22 drop freezing experiments (727 droplets total). A best-fit line to the values of *K*(*T*) obtained from the Milli-Q® purified water, was used to estimate background nuclei concentrations and was substracted from the cumulative nucleus spectra obtained later when nucleants were added. 68% confidence intervals constructed about this line were used to estimate uncertainties due to the variation in the number of these background nuclei, and this was propagated into the uncertainty associated with the cumulative nuclei spectra of the added nucleants.

## Author Contributions

D.O.S. conducted the ice nucleation experiments, analysed the data and was authored the manuscript. B.J.M. and M.E.W. oversaw the project and helped to write the manuscript. J.F.R. performed and analysed data from the size exclusion chromatography. T.F.W. and N.S.U. provided data for the background ice nucleating activities of Milli-Q water. H.C.P. and J.D.A. aided with the error analysis of the data and assisted with drafting the manuscript.

## Supplementary Material

Supplementary Information Supplementary Info

## Figures and Tables

**Figure 1:**
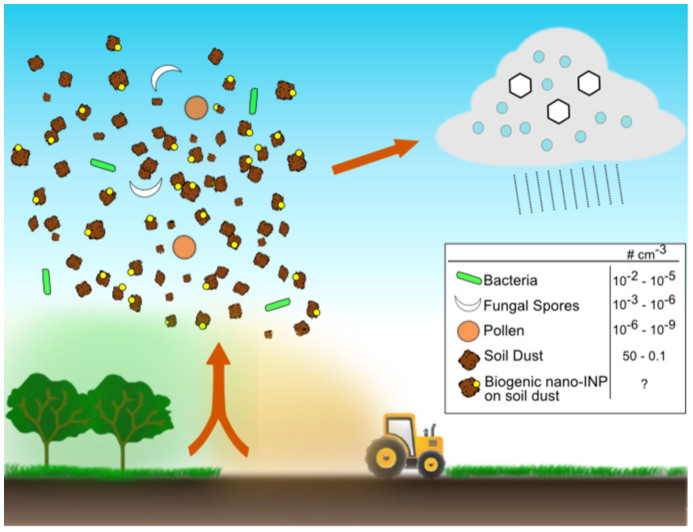
Illustration of the hypothesis that nanometre-scaled INPs of biological origin may become lofted to the atmosphere in association with soil dust particles and impact cloud glaciation. Nano-INPs associated with soil dust particulates could potentially greatly outnumber intact bioaerosol particles such as pollen, fungal spores and bacteria and therefore be much more important for ice nucleation in clouds, but are not presently represented in models. The quoted particle concentrations are zonal annual means from the simulations of Hoose et al.^45^ for an altitude corresponding to 600 hPa.
