# Supplementary material for: The relevance of nanoscale biological fragments for ice nucleation in clouds
Source: Sci Rep. 2015 Jan 28;5:8082. doi: 10.1038/srep08082 (PMC4308702; doi:10.1038/srep08082)
Supplement: Supplementary Information — Supplementary Info [file srep08082-s1.pdf]

Supplementary Information For:

The relevance of nanoscale biological fragments for ice nucleation in clouds

D. O'Sullivan, B. J. Murray, J. F. Ross, T. F. Whale, H. C. Price, J. D. Atkinson, N.S. Umo & M.E. Webb

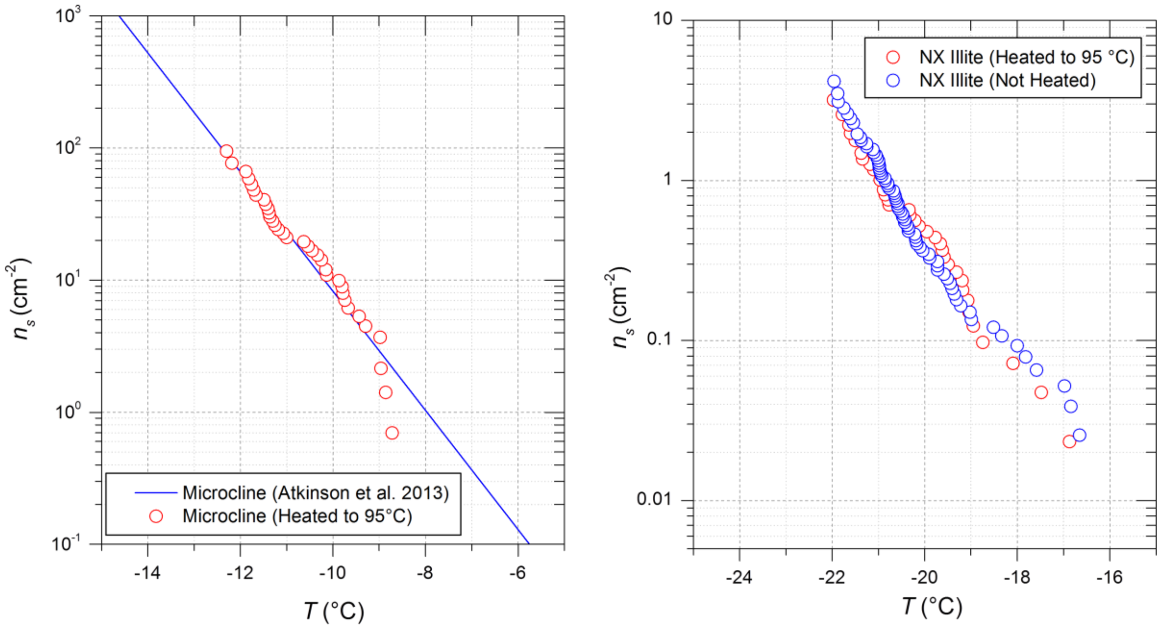

Figure S1: Ice active surface site densities for potassium feldspar (microcline) and NX illite before and after heating of the suspensions to 95°C for 45 minutes. The parameterization for unheated microcline is taken from Atkinson et al. [1], where full details of the mineralogy for both samples used can be found.

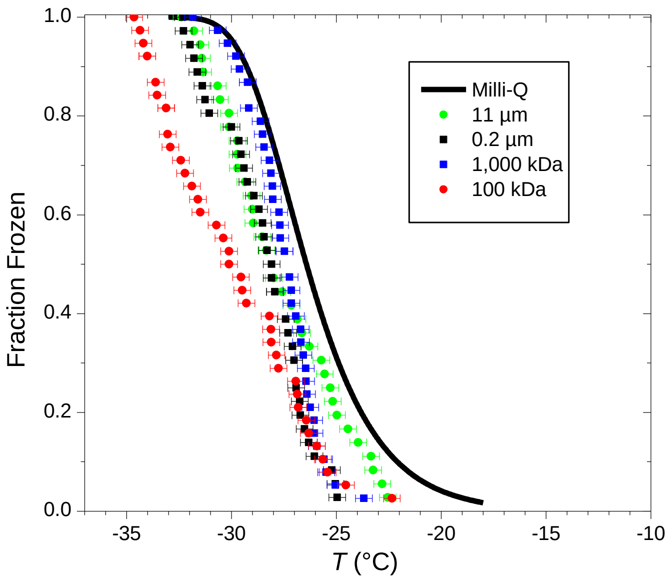

Figure S2: Fraction frozen data for Milli-Q water after it has been passed through the filters used in the following study. Illustrated are data for the 11 µm, 0.2 µm 1000 kDa and 100 kDa filters employed. Also shown is the fraction frozen curve for Milli-Q water derived from the best fit line to the cumulative nucleus spectra of 23 separate experiments (737 drops in total).

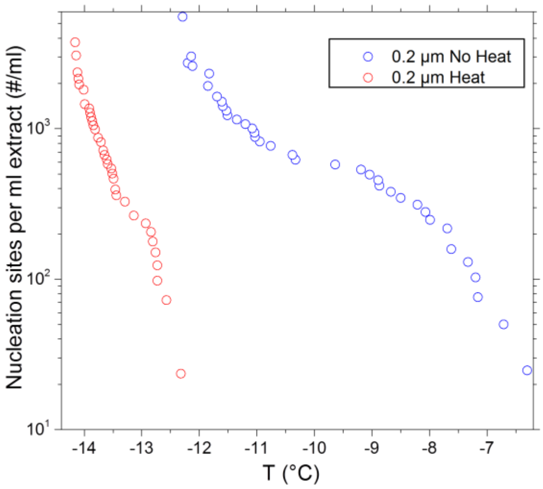

Figure S3: Cumulative nucleus spectra for the 0.2 µm a filtrates of the soil suspension. Indicated are the samples prior to heating (blue circles) and after heating (red circles)

1 Atkinson, J. D. *et al.* The importance of feldspar for ice nucleation by mineral dust in mixed-phase clouds. *Nature* **498**, 355-358, (2013).
